# Ferromagnetism induced by entangled charge and orbital orderings in ferroelectric titanate perovskites

**DOI:** 10.1038/ncomms7677

**Published:** 2015-03-25

**Authors:** N. C. Bristowe, J. Varignon, D. Fontaine, E. Bousquet, Ph. Ghosez

**Affiliations:** 1 Theoretical Materials Physics, University of Liège, B-4000 Sart Tilman, Belgium; 2 Department of Materials, Imperial College London, London SW7 2AZ, UK

## Abstract

In magnetic materials, the Pauli exclusion principle typically drives anti-alignment between electron spins on neighbouring species resulting in antiferromagnetic behaviour. Ferromagnetism exhibiting spontaneous spin alignment is a fairly rare behaviour, but once materialized is often associated with itinerant electrons in metals. Here we predict and rationalize robust ferromagnetism in an insulating oxide perovskite structure based on the popular titanate series. In half-doped layered titanates, the combination of Jahn–Teller and oxygen breathing motions opens a band gap and creates an unusual charge and orbital ordering of the Ti *d* electrons. It is argued that this intriguingly intricate electronic network favours the elusive inter-site ferromagnetic (FM) ordering, on the basis of intra-site Hund's rules. Finally, we find that the layered oxides are also ferroelectric with a spontaneous polarization approaching that of BaTiO_3_. The concepts are general and design principles of the technologically desirable FM ferroelectric multiferroics are presented.

## Abstract (web summary)

Magnetic insulators often display antiferromagnetic ordering owing to implications from the Pauli exclusion principle. Here, the authors predict ferromagnetism on the basis of intra-site Hund's rules in ferroelectric titanate superlattices showing charge and Jahn–Teller induced orbital orderings.

## Graphical abstract

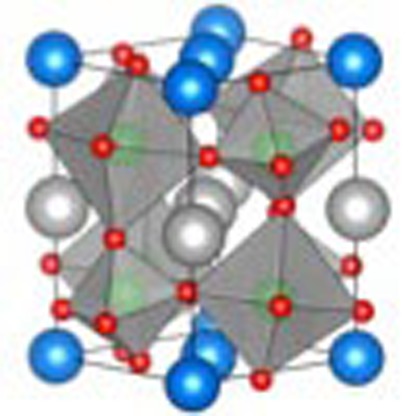

Perovskite oxides exhibit a fascinating range of physical properties, including ferroelectricity, (anti)ferromagnetism (AFM), superconductivity and magnetoresistance. This diverse behaviour is appealing for both fundamental and applied investigations, and has resulted in an intense global research effort over the past few decades. Many of these functional properties manifest due to the complex and subtle interplay between spin, charge, orbital and lattice degrees of freedom in perovskites^1^,^2^,^3^,^4^. Of the perovskites, the doped manganites have become a prototypical playground for the study of this interplay. Just considering the case of half-doping, that is, 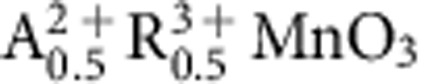, where A^2+^ is a divalent alkaline earth metal ion and R^3+^ is a trivalent rare earth ion, manganites exhibit a rich variety of electronic phases. For example, half-doped manganites can display ferromagnetic (FM) or A-type AFM metallic behaviour^5^,^6^,^7^ or more commonly a CE-type AFM Mott insulating phase^8^,^9^ associated with two different charge orderings (rocksalt^10^ and columnar^11^) and two different orbital orderings (‘ferro’ and ‘antiferro’ Mn *d e*_*g*_ orderings^12^). The preferred electronic phase appears to be strongly dependent on the A^2+^ and R^3+^ cation sizes and whether they appear disordered (such as with Ca and La/Pr) or layered (such as for Ba and La/Tb/Y^5^,^10^,^12^) in the crystal.

In this regard, it is interesting to compare the physics of the half-doped manganites, with that of the half-doped titanates. At the bulk level, the A^2+^ and R^3+^ cations are found to naturally disorder^13^,^14^ in the titanates, and typically no charge and orbital-ordered Mott insulating phase is observed at half-doping^15^. An exception has been recently discovered for the case of very small A^2+^-cations, such as Ca_0.5_Lu_0.5_TiO_3_, where a rocksalt charge-ordered and *d*_*xy*_ *t*_2*g*_ orbital-ordered Mott insulating phase was recently proposed^16^. On the other hand, in layered superlattices consisting of a repeating unit of *k* layers of A^2+^TiO_3_ with *l* layers of R^3+^TiO_3_, exotic behaviour such as an interface two-dimensional (2D) electron gas^17^, which can be FM^18^ and superconducting^19^ has been reported.

Here we consider half-doped titanates in short-period [001] superlattice form (*k*=*l*=1) (see Fig. 1), which can in principle be artificially grown using modern layer-by-layer growth techniques (see for instance refs. 20, 21 and references within). This case resembles a bulk-like double perovskite, where every Ti ion shares the same mixed environment at odds with thicker superlattices. A careful first principles investigation (see Methods) including all possible degrees of freedom reveals an unexpected FM and ferroelectric insulating ground state. The electronic structure exhibits an intricate orbital and charge ordering, which is argued to be at the heart of the observed ferromagnetism. A symmetry lowering structural distortion enabling this particular orbital ordering is also found to drive the ferroelectricity. The results appear rather general across the whole A^2+^TiO_3_-R^3+^TiO_3_ (*k*=*l*=1) series, being shared by a wide variety of combination of cations with a large variation of cationic sizes. This has allowed for universal physical principles to be rationalized and new multiferroic design guidelines to be proposed. The subtle interplay between electronic and structural degrees of freedom are compared with the manganites and novel features are highlighted.

## Results

### Ferroelectricity

**Figure 1:**
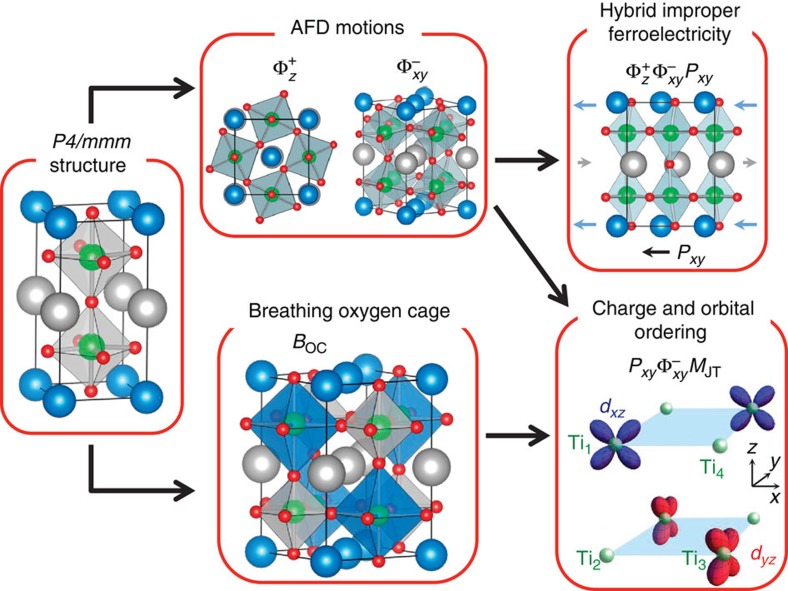
Superlattice geometry, major structural distortions, and resulting ferroelectric, charge and orbital ordering. The A^2+^TiO_3_-R^3+^TiO_3_ digital superlattice 10-atom high-symmetry tetragonal *P*4/*mmm* reference structure undergoes two major structural distortions: AFD motions and a breathing oxygen cage distortion. The rocksalt arrangement of large (blue) and small (grey) octahedral cages of the breathing distortion are shown in the 20-atom cell. The AFD motions induce ferroelectricity through a unique anharmonic coupling to an in-plane polar mode. The combination of the AFD motions and breathing oxygen cage allows for an unusual charge and orbital ordering. Blue, grey, red and green spheres represent R^3+^, A^2+^, O and Ti, respectively. Distortions are exaggerated for illustrative purposes.

To unravel the unexpected ferroelectric and FM behaviour, we begin by focussing on the atomic structure of the A^2+^TiO_3_-R^3+^TiO_3_ superlattice (see Fig. 1). Unless stated otherwise the results presented throughout, although qualitatively similar across the whole series (see Supplementary Tables 3 and 4), are presented for the case of SmTiO_3_-SrTiO_3_. In all cases, we find a *P*2_1_ symmetry ground state that consists of a complex combination of several lattice distortions (see Table 1) of the high-symmetry (*P*4/*mmm*) cube-on-cube double perovskite. Out of all the distortions, the largest in amplitude are oxygen octahedral rotations, both in-phase around the out-of-plane (*z*) axis, 
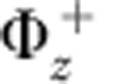
 and anti-phase around the in-plane (*x* and *y*) axes, 
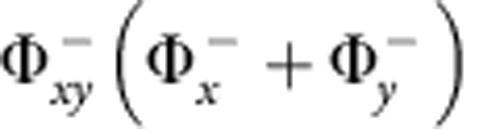
. These so-called antiferrodistortive (AFD) motions are ubiquitous in perovskites, appearing through steric effects described by the Goldschmidt tolerance factor^22^. The particular AFD pattern found here, a^−^a^−^c^+^ in Glazer’s notations^23^, is the most common pattern shown by perovskites^24^, certainly with tolerance factors between 0.8–1.0, the case studied here. This particular AFD pattern is stabilized over others in simple bulk perovskites through unique anharmonic couplings allowing the subsequent appearance of anti-polar A-cation motions^25^,^26^,^27^, located at the zone-boundary of the cubic ABO_3_ Brillouin zone. In the A^2+^TiO_3_-R^3+^TiO_3_ digital superlattices, this A- and R-cation motion transforms to the zone-centre, becoming polar in nature. The precise form of the anharmonic coupling is trilinear, 
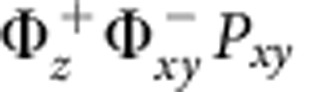
, which is the signature of the so-called rotationally driven hybrid improper ferroelectricity^28^,^29^,^30^,^31^,^32^. Indeed, we calculate all superlattices to exhibit spontaneous polarizations in the range of 7–23 *μ*C cm^−2^ (see for example Table 1), approaching that of BaTiO_3_, which is among the highest reported in hybrid improper ferroelectrics. Since the *P*_*xy*_ mode has a ‘ferri’-like character of the A-and R-cation motion (see Fig. 1), the large polarization is achieved by maximizing the mode polarity through choosing A- and R-cations not only with asymmetric cation sizes^33^ but also here thanks to significantly distinct Born effective charges (or valences in the simplest picture).

### Charge ordering

A necessary requirement of ferroelectricity is to be electronically insulating, which is not *a priori* obvious in these half-doped titanates. Allowing only AFD motions yields a metastable system with the usual *Pmc*2_1_ symmetry (equivalent to *Pnma* but for the superlattice). Within this symmetry, the system is metallic and all Ti ions share the same magnetic moment by symmetry (roughly 0.45 *μ*_B_ per Ti site). However, we observe an electronic instability leading to an incipient charge ordering between the four Ti sites. Indeed, releasing the symmetry constraint on the electronic wavefunction while keeping the atoms fixed to a *Pmc*2_1_ structure produces an energy gain due to an incipient *d*^1^–*d*^0^ charge ordering. Relaxing the geometry produces the *P*2_1_ ground state with the appearance of a related breathing *B*_OC_ motion, which expands or contracts the oxygen octahedra in nearest neighbour unit cells (see Fig. 1 and Table 1). This breathing distortion does not lift the degeneracy of the *t*_2*g*_ levels of the Ti atoms at the centre of each octahedra, but does lift the degeneracy between Ti sites, amplifying the charge ordering, and helping render the superlattices insulating. The charge ordering mimics the rocksalt pattern, and hence appears at the *R-*point (*M*-point) of the cubic (tetragonal) Brillouin zone, maximizing the distance between the more highly charged *d*^0^ Ti^4+^ ions. Please see Supplementary Note 1, and associated Supplementary Tables 6 and 8, for further discussion on the breathing mode and charge ordering.

### Orbital ordering

**Figure 2:**
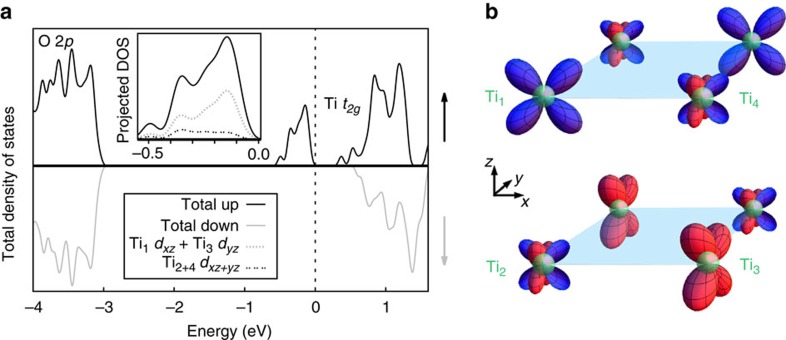
Electronic structure–charge and orbital ordering. (**a**) Total and projected spin-polarized DOS. The upper panel (solid black), and lower panel (solid grey) correspond to up and down spins, respectively. The vertical dashed line represents the Fermi level. The Projected DOS in the inset compares the Ti_1_ *d*_*xz*_+Ti_3_ *d*_*yz*_ (grey fine dashed lines) with the Ti_2+4_ *d*_*xz*+*yz*_ (black dashed lines) DOS of the spin-split-off state. (**b**) Sketch of the resulting orbital ordering. Green spheres represent Ti sites, and red and blue lobes represent *d*_*yz*_ and *d*_*xz*_ orbitals, respectively.

This charge-ordered insulating state is indeed found, as indicated through the spin-resolved density of states (DOS), as presented for the FM solution in Fig. 2 (for the full projected DOS see Supplementary Fig. 1). The states near the Fermi level exhibit Ti *d* character, while the O 2*p* states appear deeper into the valence. A band gap separates an occupied spin-polarized ‘split-off’ band from the remaining unoccupied Ti *d* conduction band. This ‘split-off’ valence is found to consist of 2 bands with the majority of weight located at 2 different Ti sites, out of the 4 possible Ti sites in total in the 20-atom unit cell (see Fig. 1). These two sites are surrounded by the expanded oxygen octahedra, which we label the Ti *d*^1^ sites, as opposed to the two other Ti *d*^0^ sites surrounded by a contracted oxygen octahedra. Interestingly the orbital occupation of the two *d*^1^ sites are different with one showing *d*_*xz*_ and the other *d*_*yz*_ character. The resulting orbital ordering corresponds to the same pattern, *albeit* with half the sites empty, as that achieved through a Jahn–Teller distortion appearing at the *M*-point of the cubic Brillouin zone (*M*_JT_). This lattice distortion is indeed observed in the ground state (see Table 1). In fact it is found that the AFD motion themselves can achieve this orbital ordering, in absence of the *M*_JT_, by lowering the symmetry of the bulk (superlattice) cubic (tetragonal) reference phase to orthorhombic (monoclinic). Indeed, similarly to the improper appearance of the polar A-cation motions, the AFD motions also drive the appearance of the *M*_JT_ distortion through another trilinear coupling, 
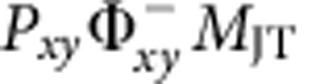
34. The fact that the AFD motions produce the same orbital ordering as the *M*_JT_ distortion is simply a consequence of this symmetry relation.

Interestingly, we find that the charge ordering is not exactly the ideal case of *d*^1^–*d*^0^. Defining *m* as the magnitude of the local magnetic moment, we find a *d*^(1−*m*)^–*d*^*m*^ ordering, with a significant weight on the nominally *d*^0^ sites (0.11<*m*<0.18 depending on the A- and R-species), displaying a mixed *d*_*xz*_–*d*_*yz*_ character. The system remains insulating despite this delocalized state, through a strong hybridization of the two occupied Ti *d* bands. The resulting intricate and entangled charge and orbital ordering is sketched in Fig. 2.

### Magnetic ordering

**Figure 3:**
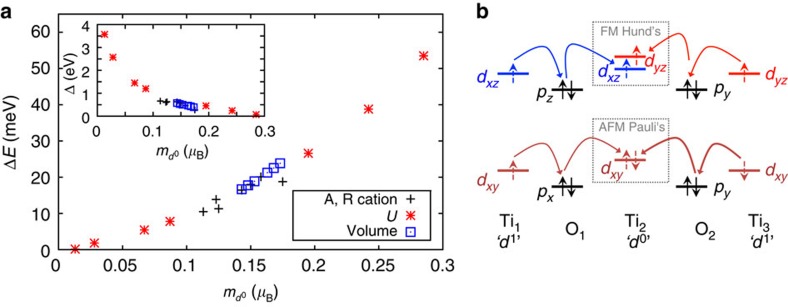
Insights into magnetic interactions. (**a**) Energy gain of FM compared with AFM state (see Methods) per 20-atom formula unit, Δ*E*, as a function of the moment on the *d*^0^ sites, *m*_*d*_0. Inset: band gap Δ versus *m*_*d*_0. The different points correspond to various perturbations of the superlattice, including varying the A- and R-cation species, the on-site Coulomb repulsion *U* and the volume of the cell. (**b**) Simplified spin exchange diagram between nearest neighbour *d*^1^ sites (Ti_1_ and Ti_3_) via a *d*^0^-site (Ti_2_). The curved arrows represent inter-site spin hopping through oxygen ions, and the dashed box represents intra-site spin-exchange via Hund’s rules, in the case of *d*_*xz*_–*d*_*yz*_ orbital ordering due to AFD motions, or Pauli's exclusion principle, in the case of *d*_*xy*_ orbital ordering in the absence of AFD motions.

Having discussed the lattice, charge and orbital degrees of freedom, and their coupling, we move to the final degree of freedom—spin. Various magnetic orderings (see methods and Supplementary Tables 4 and 5) were studied, and in all cases and chemistries the FM state was unexpectedly found to be the lowest in energy. In an attempt to understand the origin of this rare insulating FM ground state, we observe a universal trend across A^2+^TiO_3_-R^3+^TiO_3_ superlattices with all A- and R-species and various applied perturbations. This trend is shown in Fig. 3, where it is seen that the energy difference between AFM and FM states, or more simply the strength of the FM exchange, is clearly strongly dependent on the spin density of *d* electrons on the nominally *d*^0^ sites. Indeed, as the system tends to the ideal charge ordering *d*^1^–*d*^0^, the FM and AFM energies tend to equilibrate. Therefore, in this regime, the spins on the two *d*^1^ sites are completely decoupled. However, as the *d*^0^-site electrons become populated, the FM exchange strengthens. This key observation indicates that the FM exchange mechanism relies on a real intra-site spin exchange on the nominally *d*^0^ sites, rather than a virtual direct exchange between *d*^1^ sites. We propose the intra-site FM spin exchange as Hund’s rule (see Fig. 3).

The explanation of the FM interaction between *d*^1^ sites relies on two simple arguments, sketched in Fig. 3 (for simplicity in the following, we only consider the four Ti sites of Fig. 2, but the same arguments hold when including all neighbours in 3D). First, due to covalency effects, the electron on the Ti_1_ (*d*^1^) site delocalizes partly on the neighbouring Ti_2_ and Ti_4_ (*d*^0^) sites necessarily with the same spin (since it is the same electron) and the analogous occurs to the electron on the Ti_3_ (*d*^1^) site. Second, due to the orbital ordering, the charges on Ti_2_ (and equivalently Ti_4_) that originate independently from Ti_1_ and Ti_3_ populate a different orbital (*d*_*xz*_ and *d*_*yz*_ respectively). In the same spirit as Hund’s rule of maximum multiplicity, which maximizes the spin of an electron configuration of an atom, a FM solution of the 
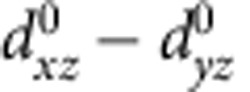
 spins becomes the ground state. This rule minimizes the intra-site Coulombic energy. The FM solution relies on both kinetic and Coulomb contributions, to delocalize the spin-polarized *d*^1^ electrons partially on the *d*^0^ sites and to align the *d*^0^ intra-site spins, respectively. In this regard, it resembles a double (kinetic+Coulomb) exchange, even if in an insulator. This FM mechanism in *d*^0^–*d*^1^ charge-ordered systems, which has been previously overlooked, extends the list of *d*^*i*^–*d*^*j*^ charge orderings considered within the Goodenough–Kanamori rules^8^,^35^,^36^.

We performed two computational experiments to further support this picture. In the first computational experiment, we took the ground state structure and artificially modified the magnitude of the *M*_JT_ distortion (leaving all other lattice modes unchanged). Eventually at a huge *M*_JT_ distortion (50 times larger than the ground state magnitude, but similar in size to the prototypical Jahn–Teller system LaMnO_3_), the 
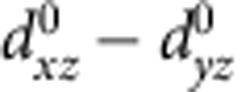
 splitting (see Fig. 3) is so large that only one of the orbitals becomes populated at each *d*_0_-site, removing the Hund’s interaction and producing an AFM ground state. In the second case, the AFD motions were artificially neglected (along with all other motions except B_OC_). The tetragonal symmetry can still lead to an insulating charge-ordered state but now with *d*_*xy*_ orbital ordering, once an unrealistically large on-site Coulomb repulsion *U* (>7 eV) is enforced^37^. We have reproduced this constraint and find that the AFM solution now becomes the ground state. This can again be understood through the intra-site spin exchange on the nominally *d*^0^ sites (see Fig. 3). An anti-alignment of spins on the intra-site 
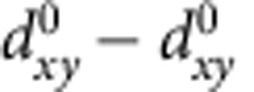
 orbitals (and hence on the inter-site 
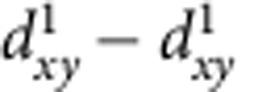
 orbitals) is favoured from Pauli’s exclusion principles—now that the same orbital (spacial coordinate) is occupied, opposite electron spins are favoured. Therefore the combination of charge ordering at the *R*-point, via breathing oxygen cage motions, and orbital ordering at the *M*-point, via AFD and *M*_JT_ motions (but not too large), of the cubic Brillouin zone are the key to realize the FM ground state.

## Discussion

We note the FM mechanism here is distinct from the FM behaviour found in some R^3+^TiO_3_ bulk compounds (for R from Gd to Yb). To illustrate, for example, we note the FM coupling becomes stronger as the A size decreases and band gap^38^ increases in bulk R^3+^TiO_3_ compounds. This is seen through a transition from AFM to FM and then a rise in FM *T*_C_ with decreasing A size^39^. The reverse trend is observed here, where the FM coupling strengthens with increasing A-cation size and decreasing band gap.

It is interesting finally to compare the FM solution of the half-doped titanates, to the (A-type or E-type) AFM solution of the half-doped manganites. The magnetic ordering is likely different due to the superexchange interaction in the manganites between the half-filled core *t*_2*g*_ electrons on every Mn site. This superexchange path is clearly cutoff by the rocksalt arrangement of *d*^0^ sites in the titanates studied here. A FM insulating phase of the manganites does exist for the specific case of Pr_1−*x*_Ca_*x*_MnO_3_ with doping *x*≈0.2 (refs 40, 41, 42), where a FM exchange wins due to a particular *e*_*g*_ orbital polaron ordering^43^, not completely dissimilar to the *t*_2*g*_ case here, using the Goodenough–Kanamori rules^8^,^35^,^36^.

Having rationalized the key concepts underlying the FM and ferroelectric behaviour, we can propose some basic design rules and suggest alternative materials to achieve a similar multiferroic state. To ensure an insulating ground state, the smaller the A- and R-cations the larger the band gap, consistent with the recent observation of insulating Ca_0.5_Lu_0.5_TiO_3_ (ref. 16). Maximizing the ferroelectric polarization can be achieved by choosing the greatest asymmetry in both the Born charge and size of the A- and R-cations. Regarding ferromagnetism, the exchange coupling constant *J*, and hence the FM *T*_C_, increases the greater the delocalization of charges onto the nominally *d*^0^ sites, which can be altered with strain and A-site chemistry as shown in Fig. 3. It could be interesting to test the same concepts on other B-cations such as the 4*d* and 5*d* transition metals, *d*^9^–*d*^10^ systems such as the cuprates, which can be viewed as a *d*^1^–*d*^0^ hole charge ordering, or *d*^6^–*d*^7^ systems with ordering of the single *e*_*g*_ electron instead of the *t*_2*g*_ in the titanates. Here we might expect a similar type of entangled charge and orbital orderings leading to unexpected ferromagnetism. We hope that this study might encourage the search for related novel electronic phases within these systems.

## Methods

### First principles calculations

Here we describe a three-step first principles strategy for the titanate calculations. (i) Initially hybrid functional calculations, using the B1WC functional^44^ within the Crystal code^45^, were performed on bulk titanates (YTiO_3_, LaTiO_3_, SrTiO_3_ and BaTiO_3_) and compared with experiment (see Supplementary Table 1). The details of the pseudopotentials and basis sets for La, Sr, Ti and O can be found in ref. 46, while for Ba and Y details can be found in refs. 47, 48 respectively. (ii) Once verified on the bulk, hybrid functional calculations were performed on several representative R^3+^TiO_3_-A^2+^TiO_3_ superlattices. Ground states were determined through condensing various lattice instabilities and recalculating phonon frequencies. Band gaps and FM and AFM energies were computed on the ground states. (iii) To make the calculations computationally tractable, and allow simulation of many more chemistries, GGA+U calculations^49^, using the PBEsol functional^50^ and projector augmented wave potentials within the VASP code^51^, were performed on the relaxed hybrid functional superlattice ground state structures. The on-site Coulomb repulsion *U* was fitted to simultaneously reproduce the band gap and spin flip energy of the hybrid functionals, and minimize atomic forces (see Supplementary Table 2), with a value of *U*=3.0 eV found to be appropriate. This allowed for full structural relaxation using GGA+U on a wide-range of R^3+^TiO_3_-A^2+^TiO_3_ structures (R^3+^: La, Pr, Sm, Y, Tm, Lu; A^2+^: Sr, Ba, Ca and we also include Eu^2+^—see Supplementary Table 7). We used a 5 × 5 × 3 Monkhorst-Pack k-point mesh to model the 20-atom cell and a plane wave cutoff of 500 eV. All lattice vectors were fully relaxed. Small to moderate in-plane strains resembling thin film epitaxy were not found to alter the qualitative findings presented. Structural optimizations were performed until the difference of forces were <10^−6^ eV Å^−1^ and the energy difference between conjugate gradient steps became lower <10^−8^ eV. Note: we do not consider R *f*-electrons explicitly to simplify calculations, since in practice the 4*f*-electrons order at much lower temperatures than the 3*d*-electrons. We have tested explicitly including *f*-electrons and find that the results are not affected. Unless stated otherwise the AFM ordering presented is the simplest AFM configuration between first-neighbour *d*^1^ sites allowed within the 
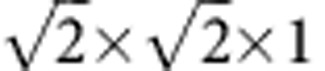
 20-atom cell. For longer range AFM orderings 2 × 2 × 1 and 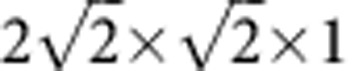, 40-atom supercells were relaxed with all possible spin orderings considered, including the CE-type. AFM orderings requiring cell doubling out-of-plane (80 atoms) were additionally checked (see Supplementary Table 5). Atomic positions were relaxed for all AFM orderings. The polarization was computed using the Berry phase approach as implemented in VASP. The amplitude *Q* (Å) of lattice distortions, was determined through the symmetry-mode analysis using the AMPLIMODES software^52^.

## Author contributions

All authors contributed extensively to the work presented in this paper.

## Additional information

**How to cite this article:** Bristowe, N. C. *et al.* Ferromagnetism induced by entangled charge and orbital orderings in ferroelectric titanate perovskites. *Nat. Commun.* 6:6677 doi: 10.1038/ncomms7677 (2015).

## Supplementary Material

Supplementary Information Supplementary Figure 1, Supplementary Tables 1-8, Supplementary Note 1 and Supplementary References.

## Figures and Tables

**Table 1:** Key quantities for a selection of A^2+^TiO_3_-R^2+^TiO_3_ superlattices.

**R**^3+^**, A**^2+^	***Q***					***P***	**Δ**	**Δ*****E***
	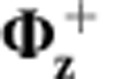	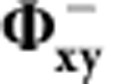	***P***_***xy***_	***B***_**OC**_	***M***_**JT**_			
Sm, Sr	0.96	1.19	0.56	0.10	0.04	14.9	0.46	20.1
Y, Sr	1.10	1.30	0.66	0.11	0.04	16.7	0.57	18.0
Tm, Sr	1.18	1.36	0.72	0.11	0.03	18.2	0.63	16.4
Sm, Ba	0.75	0.96	0.48	0.13	0.07	18.6	0.50	18.5
Y, Ba	0.95	1.08	0.59	0.14	0.07	21.2	0.60	13.9
Tm, Ba	1.05	1.16	0.65	0.16	0.07	23.4	0.66	10.5

AFD, antiferrodistortive; AFM, antiferromagnetism.

Amplitude *Q* (Å) of lattice distortions (in-phase 
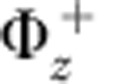
 and anti-phase 
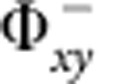
 AFD motions, polar mode *P*_*xy*_, Jahn–Teller mode appearing at the *M*-point of the cubic Brillouin zone *M*_JT_ and breathing oxygen cage *B*_OC_), polarization *P* (μC cm^−2^), band gap Δ (eV) and gain of energy for FM versus AFM solution (see Methods) per 20-atom formula unit Δ*E* (meV).
