# Supplementary material for: Ferromagnetism induced by entangled charge and orbital orderings in ferroelectric titanate perovskites
Source: Nat Commun. 2015 Mar 25;6:6677. doi: 10.1038/ncomms7677 (PMC4389245; doi:10.1038/ncomms7677)
Supplement: Supplementary Information — Supplementary Figure 1, Supplementary Tables 1-8, Supplementary Note 1 and Supplementary References. [file ncomms7677-s1.pdf]

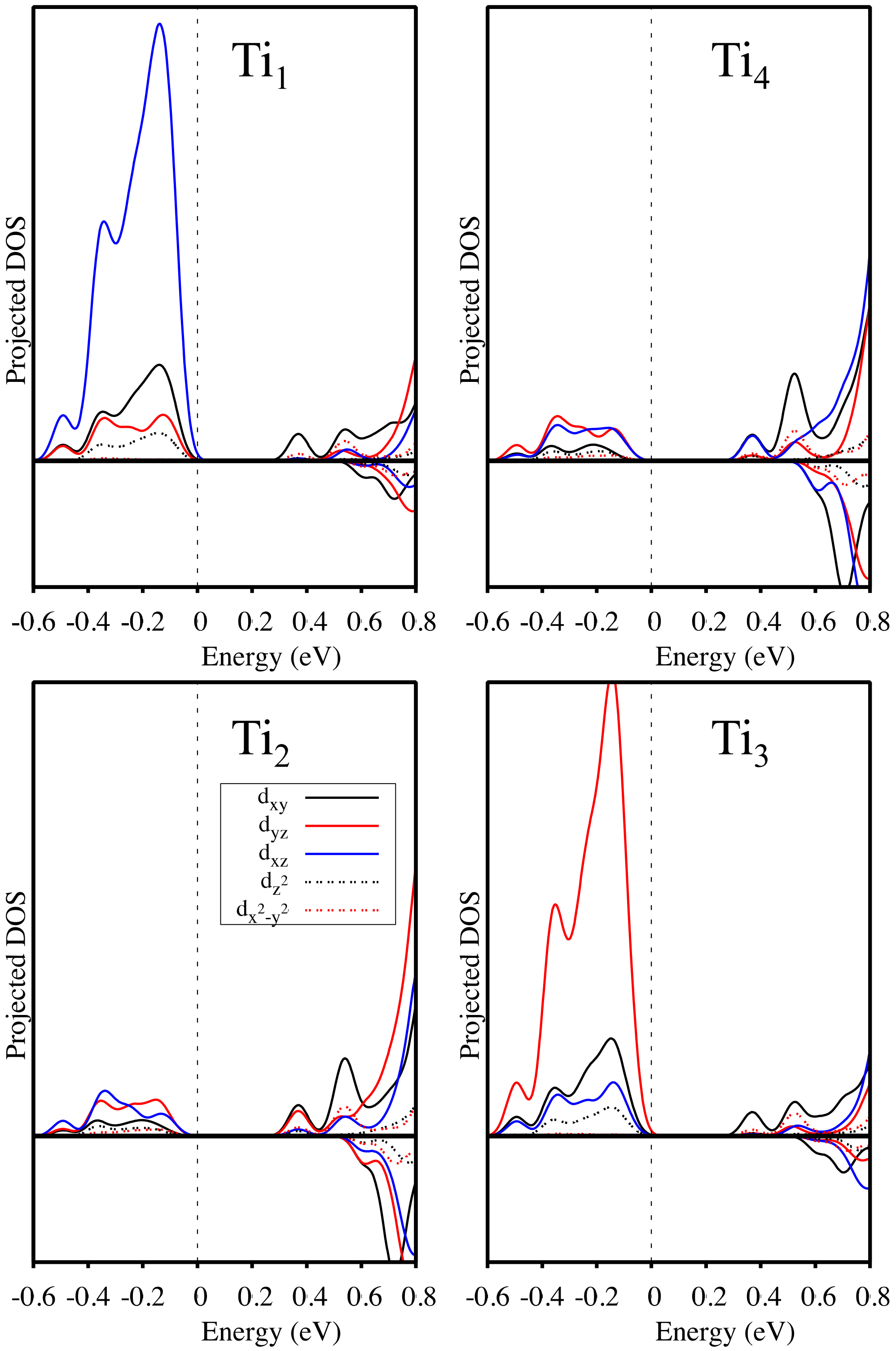

Supplementary Figure 1: Projected density of states (DOS) of the d states for the four titanium ions in the SmSr superlattice (Ti_1-Ti_4 as defined in the main text).

R	a	b	c	Φ	Θ	m	Δ	ΔE
La	5.53	7.92	5.65	8.78	11.80	0.9	1.2	-28
	5.59	*7.90*	*5.64*	*9.60*	*13.11*	*0.57*	*0.2*	*G-type AFM ground state*
Y	5.27	7.56	5.69	12.68	19.68	0.9	1.3	+70
	5.34	*7.61*	*5.69*	*12.89*	*20.06*	*0.84*	*1.2*	*FM ground state*

Supplementary Table 1: Comparison of B1WC [1] (normal font) with experiments [2,3] (given in italics) on bulk $RTiO_3$. A comparison for $ATiO_3$ (A = Sr, Ba and Ca) can be found in the original reference [1]. DFT overestimates $LaTiO_3$ Ti moment, *m* (μ_B), and band gap, Δ (eV), due to magnetic fluctuations. These fluctuations are suppressed, and hence DFT becomes in good agreement, with increasing oxygen octahedral rotations [4], Θ and Φ (°). This is why only the smaller R cations are reported in the main text. a, b and c (Å) refer to the orthorhombic lattice parameters, and ΔE (meV) is the energy of the G-type AFM solution minus the FM solution per 20-atom unit cell.

R, A	Δ	ΔE	$m(d^0)$	$m(d^1)$
Y, Sr	0.52	25.4	0.24	0.75
	0.45	*26.6*	*0.20*	*0.71*
Y, Ba	0.54	21.6	0.19	0.79
	0.46	*20.7*	*0.16*	*0.74*

Supplementary Table 2: Comparison of key quantities for B1WC [1] (normal font) vs PBEsol+U (U=3 eV) [5,6] (given in italics) for representative $ATiO_3$-$RTiO_3$ superlattices. The coordinates and lattice parameters were in both cases fixed to those of the relaxed B1WC. ΔE (meV) is the gain of energy for FM vs AFM solution per 20-atom formula unit, and $m(d^0)$ and $m(d^1)$ (μ_B) the magnetic moments of the Ti 'd^0' and 'd^1' sites.

R, A	Q				
	Φ_z^+	Φ_{xy}^-	P_{xy}	B_{OC}	M_{JT}
La, Sr	0.716	1.022	0.357	0.092	0.039
Pr, Sr	0.802	1.076	0.438	0.097	0.046
Sm, Sr	0.965	1.190	0.561	0.103	0.042
Y, Sr	1.103	1.301	0.664	0.109	0.036
Tm, Sr	1.178	1.362	0.718	0.112	0.031
La, Ba	0.306	0.716	0.194	0.094	0.044
Pr, Ba	0.483	0.817	0.311	0.109	0.062
Sm, Ba	0.753	0.956	0.476	0.126	0.069
Y, Ba	0.953	1.082	0.590	0.144	0.069
Tm, Ba	1.054	1.156	0.654	0.156	0.067
Lu, Ca	1.257	1.670	0.837	0.124	0.016

Supplementary Table 3: Amplitude Q (Å) of lattice distortions for the relaxed (using PBEsol+U) $ATiO_3$-$RTiO_3$ superlattices: in-phase $\Phi^+{}_z$ ($M_2{}^+$) and anti-phase $\Phi^-{}_{xy}$ ($M_5{}^-$) AFD motions, polar mode P_{xy} ($\Gamma^-{}_5$) , Jahn-Teller mode appearing at the M-point of the cubic Brillouin zone M_{JT} ($M_3{}^+$), breathing oxygen cage B_{OC} ($M_2{}^-$) .

R, A	P	Δ	ΔE	Z(R)	Z(A)
La, Sr	7.7	0.27	186.3*	4.31	2.62
Pr, Sr	10.4	0.40	400.5*	4.15	2.61
Sm, Sr	14.9	0.46	20.1	4.04	2.58
Y, Sr	16.7	0.57	18.0	3.90	2.55
Tm, Sr	18.2	0.63	16.4	3.84	2.54
La, Ba	7.5	0.18	214.6*	4.41	2.92
Pr, Ba	12.8	0.32	344.0*	4.32	2.87
Sm, Ba	18.6	0.50	18.5	3.99	2.89
Y, Ba	21.2	0.60	13.9	3.81	2.85
Tm, Ba	23.4	0.66	10.5	3.74	2.82
Lu, Ca	14.3	0.63	11.3	3.95	2.42

Supplementary Table 4: Physical quantities for a selection of $ATiO_3$-$RTiO_3$ superlattices including polarization, P (μC cm^{-2}), band gap, Δ (eV), gain of energy for

FM vs AFM solution per 20-atom formula unit, ΔE (meV) and A or R cation diagonal component of the Born charge computed along the polar axis, Z (electrons). * indicates the AFM solution was metallic or on the verge of metallicity.

R, A	ΔE	ΔE_1	ΔE_2	ΔE_3	ΔE_4	ΔE_5	ΔE_6	ΔE_7
Tm, Ba	10.5	4.7	7.0	4.4				
Sm, Sr	20.1	9.9	6.2	8.0				
Sm, Eu	18.5				5.7	6.1	5.7	4.4
Lu, Sr	14.0				8.2	8.3	11.3	5.4
Sm, Sr*	-0.5		-19.2	-19.2				

Supplementary Table 5: The gain of energy for the FM solution compared to various AFM solutions in units of meV per 20-atom formula unit. The two superlattices chosen represent the ones of the main text with the strongest and weakest FM solution. ΔE is, as defined above, with respect to the AFM solution allowed within the 20-atom unit cell, ΔE_1 is with respect to the new AFM solution achieved within the 40-atom pseudo-cubic cell (2×2×2 with respect to cubic 5-atom bulk cell) and ΔE_2 and ΔE_3 are with respect to the CE-type AFM solutions allowed within the 40-atom $2\sqrt{2}\times\sqrt{2}\times2$ cell where the longest axis or shortest axis is aligned with the polar direction respectively. Finally 80-atom unit cells ($2\sqrt{2}\times\sqrt{2}\times4$ with respect to cubic 5-atom bulk cell) were constructed with pseudo T and S AFM orderings [7]. Again the longest axis (ΔE_4 and ΔE_5 for T and S respectively) or shortest in-plane axis (ΔE_6 or ΔE_7 for T and S respectively) can be aligned with the polar direction for each ordering. *indicates the calculation was performed without AFD motions, only allowing for the breathing Jahn-Teller to appear. As described in the main text, for a sufficiently large on-site Coulomb repulsion U (here we report for U=5.5eV) the system remains insulating with d_{xy} orbital ordering.

R, A	$m(d^0)$	$m(d^1)$	$Z(d^0)$	$Z(d^1)$	$q(d^0)$	$q(d^1)$
La, Sr	0.175	0.714	6.26	3.28	1.68	1.77
Pr, Sr	0.169	0.733	6.09	3.36	1.68	1.78
Sm, Sr	0.158	0.744	5.99	3.40	1.67	1.78
Y, Sr	0.150	0.754	5.86	3.44	1.67	1.77
Tm, Sr	0.143	0.759	5.80	3.46	1.67	1.78
La, Ba	0.181	0.709	6.43	3.28	1.68	1.77
Pr, Ba	0.165	0.735	6.11	3.55	1.67	1.77
Sm, Ba	0.143	0.757	6.03	3.61	1.66	1.77
Y, Ba	0.123	0.773	5.99	3.73	1.65	1.77
Tm, Ba	0.113	0.783	5.87	3.80	1.65	1.77
Lu, Ca	0.125	0.773	5.90	3.51	1.67	1.78

Supplementary Table 6: Evidence for inequivalence of Ti ‘d^1’ and ‘d^0’ sites, given by the magnetic moments, *m* (μ_B), the average of the diagonal components of the Ti Born effective charge, *Z* (electrons), and the d electron LDA+U occupations, *q*.

R^{3+}	P	ΔE	$m(d^0)$	$m(d^1)$
Pr	7.8	16.8	0.166	0.722
Sm	10.7	18.5	0.156	0.732
Tm	13.8	16.6	0.146	0.745

Supplementary Table 7: Extension of results to the case of $EuTiO_3$-$RTiO_3$ superlattices where Eu^{2+} acts as the A-cation. Polarization, *P* (μC cm^{-2}), gain of energy for FM vs AFM solution per 20-atom formula unit, Δ*E* (meV) and magnetic moments, *m* (μ_B), as before.

Ti	spin	xy	xz	yz	x^2-y^2	z^2	Total
1	$\uparrow$	0.188	0.550	0.144	0.180	0.204	1.266
'd_1'	$\downarrow$	0.073	0.063	0.071	0.165	0.162	0.534
	$\uparrow + \downarrow$	0.261	0.613	0.215	0.344	0.366	1.800
	$\uparrow - \downarrow$	0.116	0.487	0.072	0.015	0.042	0.732
2	$\uparrow$	0.133	0.163	0.156	0.232	0.243	0.927
'd_0'	$\downarrow$	0.110	0.107	0.105	0.222	0.229	0.773
	$\uparrow + \downarrow$	0.243	0.270	0.261	0.454	0.472	1.700
	$\uparrow - \downarrow$	0.024	0.055	0.051	0.010	0.014	0.154
3	$\uparrow$	0.188	0.144	0.550	0.180	0.204	1.266
'd_1'	$\downarrow$	0.073	0.071	0.063	0.165	0.162	0.534
	$\uparrow + \downarrow$	0.261	0.215	0.613	0.344	0.366	1.800
	$\uparrow - \downarrow$	0.116	0.072	0.487	0.015	0.042	0.732
4	$\uparrow$	0.133	0.156	0.163	0.232	0.243	0.927
'd_0'	$\downarrow$	0.110	0.105	0.107	0.222	0.229	0.773
	$\uparrow + \downarrow$	0.243	0.261	0.270	0.454	0.472	1.700
	$\uparrow - \downarrow$	0.024	0.051	0.055	0.010	0.014	0.154

Supplementary Table 8: LDA+U d-electron occupations for the four Ti atoms in $SmTiO_3$-$SrTiO_3$. Whilst the spin density shows strong ordering, the total density less so. As can be seen, whilst the 'd^0' have a lower t_{2g} occupancy, they nearly make up for the total charge through gaining significant (non-spin polarised) weight from the e_g compared to the 'd^1' sites. This is due to the strong hybridisation of the e_g with the neighboring O 2p in the contracted octahedra around the 'd^0' sites.

Supplementary Note 1

The term “charge ordering” throughout the main article is used in the traditional sense as applied to, for example, the manganites, nickelates, ferrites and vanadates where a metallic to insulating transition is observed accompanied by the appearance of two unique transition metal sites, commonly thought to show two distinct valence states. The actual degree of charge ordering is ill-defined, depending on the non-unique method of partitioning charge amongst species, but various estimates typically suggest minimal charge disproportionation actually occurs in these materials (see for example the discussion within reference [8]). Similarly we calculate using Mulliken populations or LDA+U occupations the difference of charge to be only approximately 0.1 electrons between the two Ti sites. In this regard, it might be more accurate to replace “charge ordering” by “spin-density ordering” within this article. The inequivalence between the two Ti sites here is not only seen through the magnetic moment and orbital occupations, but also the Born effective charges which are reported here in Supplementary Tables 6 and 8.

Finally we would like to comment on the nature/origin of the associated breathing oxygen cage. Intuitively, there might be a balance between the energy cost typically associated to the activation of the B_{OC} motion in the parent compounds and the energy gain associated to the electronic reorganization it produces in the superlattice. We have run additional calculations to confirm this picture. Taking a representative superlattice, we first relaxed a $Pmc2_1$ reference configuration (without B_{OC} motion, but keeping the AFD, M_{JT} and P_{xy} modes), imposing this symmetry for both the atomic and electronic configuration, and observe no charge ordering. Keeping the atoms fixed in this $Pmc2_1$ reference structure but removing the symmetry constraint for the electrons, lowers the energy of the system by 15 meV, and allows for a charge ordering on the Ti sites even in absence of the B_{OC} atomic motion. This confirms that the charge ordering is a purely electronic instability that tends to break spontaneously the symmetry of the $Pmc2_1$ phase. Then the charge ordering is further stabilized through the B_{OC} ionic relaxation. It is therefore primarily an electronic effect further stabilized by the relative softness of breathing oxygen motions in perovskites.

Supplementary References:

[1] D. Bilc, R. Orlando, R. Shaltaf, G.-M. Rignanese, J. Iniguez, and P. Ghosez, Phys. Rev. B 77, 165107 (2008).
[2] M. Cwik, T. Lorenz, J. Baier, R. Muller, G. Andre, F. Bouree, F. Lichtenberg, A. Freimuth, R. Schmitz, E. Muller-Hartmann, et al., Phys. Rev. B 68, 060401 (2003).
[3] J. Hester, K. Tomimoto, H. Noma, F. Okamura, and J. Akimitsu, Acta Crystallogr. B 53, 739 (1997).
[4] E. Pavarini, S. Biermann, A. Poteryaev, A. Lichtenstein, A. Georges, and O. Andersen, Phys. Rev. Lett. 92, 176403 (2004).
[5] A. Liechtenstein, V. Anisimov, and J. Zaanen, Phys. Rev. B 52, 5467 (1995).
[6] J. P. Perdew, A. Ruzsinszky, G. I. Csonka, O. A. Vydrov, G. E. Scuseria, L. A. Constantin, X. Zhou, and K. Burke, Phys. Rev. Lett. 100, 136406 (2008).
[7] G. Giovannetti, S. Kumar, D. Khomskii, S. Picozzi, and J. van den Brink, Phys. Rev. Lett. 103, 156401 (2009).
[8] Y. Quan, V. Pardo, and W. E. Pickett, Phys. Rev. Lett. 109, 216401 (2012).
